# Facts and Philosophy of Dental Progress

**Published:** 1867-05

**Authors:** 


### AMERICAN JOURNAL

OF

### DENTAL SCIENCE.

Vol. I. THIRD SERIES.—MAY, 1867. No. 1.

### ORIGINAL COMMUNICATIONS.

### ARTICLE I.

### *Facts and Philosophy of Dental Progress.* No. 1.

By Professor Austen.

To the Alumni of the Baltimore College of Dental Surgery :—Gentlemen,—These papers are addressed to you, not because the subject to be treated has an interest limited to yourselves, but because my manner of treatment will be somewhat didactic. Men older than I in years and in professional experience might naturally resent a style of address suitable to the lecture room. But you who have been my pupils can bear with me, if I at times appear dogmatic. You will remember also that now, equally as when students, you are not to accept facts unless you find them to be true, nor inferences unless logically correct, and that any method or practice in art is to be adopted only when proved to be best, and retained no longer than it continues to be so. You will bear in mind, what I have always taught, that an unreasoning adoption of dogmas, formulas and practices is fatal to progress ; that it is a duty, from which no student can be excused, to increase the sum of human knowledge, by adding to ideas and experiences received, thoughts and an experience of his own.

I address these papers to you also, because they are designed to some extent, as supplemental to the teachings of past years—the *addenda* to some, the *corrigenda* to others. Science is guarded by a vigilant police who compel its teachers, like "little Joe" to "move on." They who by standing still obstruct the avenues of knowledge must turn aside or be trodden down. TRUTH is fixed and immutable: man's KNOWLEDGE thereof is progressive and changing. The lecturer is as much a student as the veriest tyro, necessarily from year to year modifying his instructions, and never so untrustworthy a teacher, as when he arrogates for his perceptions of truth that infallibility which belongs alone to the truth itself. That I should to day advise you not to do or believe some things, which in 1849 or 1859 I laid down as rule and practice, is a consequence of that DENTAL PROGRESS, the true principles and results of which will be the purpose of these papers to unfold.

Progress is literally a "going forward" we have this idea modified in the terms advancement, improvement, growth and increase. Motion towards some end is a necessity of our nature. As we advance in life we improve in skill, grow in wisdom, increase in knowledge, or else we progress downward—there is no standing still in moral, mental or artistic culture. So it is in Art and Science, but with important differences. The Man is a unit, ever increasing in power or else in weakness, growing wiser or else more ignorant, improving in virtue or progressing in crime, and the limit of a life completes (as to this world) the work of progress in either direction. But Science and Art are composite and in some sense immortal: individuals and generations contribute their share in the advancement of each, but they may also do much to retard.

The Individual may destroy himself or irreparably impair his physical, mental or moral organization: but Science is not thus at the mercy of any one man, or generation, and may fully recover from the injury inflicted by an erratic genius, a sluggish age, or a false philosophy. On the other hand, no living man of the nineteenth century has any greater capability of moral, mental or physical growth, than had our first parents: he can never become more upright than JOB, more wise than SOLOMON or stronger than SAMPSON. Man then is the same to day (with certain impairments, which are irrelevent to the present subject) as on the day when he was created. God's universe and its laws are the same in every generation But man's knowledge, of that universe and comprehension of those laws are never the same in any age. Hence the knowledge of law which we call SCIENCE; the finding out things which have existed from creation, which we call DISCOVERY; the putting together of things and laws which we call INVENTION ; and the application of science, discovery, invention and skill to the various purposes of life which we call ART—will continue so long as truth lives, and there are finite minds to search it out, and they will be, in their very essence, progressive.

Progress in science and art, then, extends through generations ; it may move slowly, run in set channels, or be misdirected in some past age, yet in a subsequent one, make rapid and harmonious progress, perhaps to be obscured by a succeeding era of barbarism . We are accustomed to regard the present as peculiarly the age of Civilization and of Progress. And so, in many respects, it unquestionably is. But events are occurring around us calculated to awaken grave doubts—as to whether existing governments are in fact "the best the world ever saw" and whether modern Christianity is really any improvement upon that of the first century. In the domains of Art, Science and Literature, this much vaunted progress is on some points more than questionable, and scarcely justifies the braggadocio spirit of the age.

The much vaunted blessing of the art of printing has an attendant curse in the demoralizing influence of what we call "the press:" and the advantage of much *reading* is offset by the disadvantage of little *thinking*. The nineteenth century has produced no poet greater than Homer, no moralist purer than Socrates, no logician superior to Aristotle: and even an American historian cannot condense into the same space more lies than did Heroditus. Architecture feebly copies the chaste models of the age of Pericles, or the grand structures of what we protestants term the Dark Ages. We try to imitate the power and beauty of Roman sculpture and mediæval painting; and confess our weakness by boasting that we can stain a piece of glass nearly as well as they did 1000 years ago, and can make pottery almost as wonderful as Palissy's. We spend our ingenuity in conjecturing how the immense stones of Balbec and Palmyra were set to place, with such marvelously accurate jointings, and we puzzle over the pyramids and monoliths of Egypt. All this too, in an age, which is eminently artistic and inventive. Clearly then there is reason to fear that, as in the days of Solomon, the world is but repeating itself.

And yet there are things "new under the sun." There is such a thing as progress, in its truest sense. Puritan hypocrisy and sectarian intolerance are signs of degeneracy: but Christianity regarded merely as a system of moral philosophy is immeasurably superior to all preceding systems by virtue of its distinguishing precept "to love and forgive:" and no argument of infidelity can rob it of this glory. The Vandals of the fifth century swept away Greek and Roman civilization; but they introduced that reverence for woman which, next to Christianity, has done most to place modern civilization so far in advance of those which were swept away.

Lord Bacon could reason no more accurately than Aristotle; but by establishing the laws of induction and connecting the reasoning with the perceptive faculties of man he changed the "syllogism," from being a plaything in the hands of the Schoolmen, into a mighty instrument for the discovery of truth. Our dwellings are not so grand as a

Grecian temple or a Gothic pile; but they are vastly more comfortable to live in. The light that comes through plain glass is not so gorgeous as if shed from a mediæval window, but it is far more useful and much more wholesome. This common, cheap thing which almost any poor man can buy, a 12 light 8 x 10 window, is in fact one of the most remarkable examples of the *unacknowledged* blessings given to us by the progress of modern civililization. We are surrounded by many such, but failing to recognize them, are found boasting of matters in which we are really far behind former ages.

Science is full of such blunders, suffering great truths to lie undetected and mistaking novelty for progress. All arts are filled with them; the dentist must carefully guard against them. To give examples, merely one of each kind, would fill volumes. I will state only one or two, taken from the history of medicine, and useful because of the practical lesson which may be drawn from them.

Paracelsus discovered in mercury a remedy of wonderful power, greater perhaps than is possessed by any single medicine. And yet it is no exaggeration to say that physicians since his day have by its means inflicted upon the human race tenfold more misery and suffering than it ever relieved. The discovery of Paracelsus was true progress; the uses made of it a most sad degeneration. But again, Hippocrates, the "father of medicine," nearly twenty-four hundred years ago, based all successful practice upon experience or experiment—that is, upon *empiricism;* for such is the precise meaning of the word which is now used as synonomous with charlatanism or quackery, to distinguish it from an educated or rational practice. Now of all systems of medical practice, the rational is the most *ir*-rational. With a materia medica made up of remedies discovered by empiricism, it looses most of the good to be derived therefrom by administering them in a "rational" way. A quack nostrum will do less harm and more good in the long run, than an apothecary shop dispensed according to some elaborate theory as to *how* and *why* medicines do and should act.

The reluctance to admit our inability to explain the action of remedies, has been the plague-spot of medicine. Reason is worse than useless in therapeutics, except as it enables us to classify our experiments and make logical deductions therefrom. After more than two thousand years of rationalism, medical men are beginning to find that in surrendering EMPIRICISM to quacks they had given up the only true flag under which the "Art of Healing" can marshal its followers. The empiricism of Hippocrates was true progress; the rationalistic method of succeeding ages was a dangerous falling back, perpetually interfering with the usefulness of grand discoveries. Harvey's discovery of circulation made surgery both less painful and less fatal; but it is more than doubtlul if the theories of Cullen, Brown or Broussais helped to make the treatment of fever more successful than in the days of Galen.

These instances may teach the dental student two valuable lessons. A remedy, an operation or an appliance shall be useful within certain limits, and its introduction an evidence of true progress; yet, pushed beyond its legitimate use, it shall become an injury to both the art and the patient. Secondly, the value of any apparatus, process, material, or practice must not be measured by *arguments* urged in its favor. Everything that does not appeal directly to our five senses, must rest upon the testimony of past experience, or await the issue of experience yet to come. I shall have occasion, in subsequent papers, to show you that a disregard of these simple rules has greatly retarded the progress of dentistry.

You have heard and read much of the rapid progress of Dental Science. I propose in the next paper to inquire into the causes of this rapidity. We shall see how far it is peculiar to it—how far common to all art and science. We shall expect to learn from this inquiry the secret of progress, and may perhaps discover what constitutes the true character and dignity of Dentistry.

